# Pharmaceutical Venous Thrombosis Prophylaxis in Critically Ill Traumatic Brain Injury Patients

**DOI:** 10.1089/neur.2021.0037

**Published:** 2022-01-07

**Authors:** Jilske A. Huijben, Dana Pisica, Iris Ceyisakar, Nino Stocchetti, Giuseppe Citerio, Andrew I.R. Maas, Ewout W. Steyerberg, David K. Menon, Mathieu van der Jagt, Hester F. Lingsma

**Affiliations:** ^1^ Center for Medical Decision Sciences, Department of Public Health, Erasmus MC–University Medical Center Rotterdam, Rotterdam, The Netherlands.; ^2^ Department of Pathophysiology and Transplants, University of Milan, Milan, Italy.; ^3^ Fondazione IRCCS Ca’ Granda, Ospedale Maggiore Policlinico, Milan, Italy.; ^4^ School of Medicine and Surgery, University of Milan-Bicocca, Milan, Italy.; ^5^ Neurointensive Care, San Gerardo Hospital, ASST-Monza, Monza, Italy.; ^6^ Department of Neurosurgery, Antwerp University Hospital and University of Antwerp, Edegem, Belgium.; ^7^ Department of Biomedical Data Sciences, Leiden University Medical Center, Leiden, The Netherlands.; ^8^ Division of Anaesthesia, University of Cambridge, Addenbrooke's Hospital, Cambridge, United Kingdom.; ^9^ Department of Intensive Care Adults, Erasmus MC–University Medical Center Rotterdam, Rotterdam, The Netherlands.

**Keywords:** intensive care units, traumatic brain injuries, venous thrombosis

## Abstract

The aims of this study are to describe the use of pharmaceutical venous thromboembolism (pVTE) prophylaxis in patients with traumatic brain injury (TBI) in Europe and study the association of pVTE prophylaxis with outcome. We included 2006 patients ≥18 years of age admitted to the intensive care unit from the CENTER-TBI study. VTE events were recorded based on clinical symptoms. Variation between 54 centers in pVTE prophylaxis use was assessed with a multi-variate random-effect model and quantified with the median odds ratio (MOR). The association between pVTE prophylaxis and outcome (Glasgow Outcome Scale-Extended at 6 months) was assessed at center level with an instrumental variable analysis and at patient level with a multi-variate proportional odds regression analysis and a propensity-matched analysis. A time-dependent Cox survival regression analysis was conducted to determine the effect of pVTE prophylaxis on survival during hospital stay. The association between VTE prophylaxis and computed tomography (CT) progression was assessed with a logistic regression analysis. Overall, 56 patients (2%) had a VTE during hospital stay. The majority, 1279 patients (64%), received pVTE prophylaxis, with substantial between-center variation (MOR, 2.7; *p* < 0.001). A moderate association with improved outcome was found at center level (odds ratio [OR], 1.2 [0.7–2.1]) and patient level (multi-variate adjusted OR, 1.4 [1.1–1.7], and propensity adjusted OR, 1.5 [1.1–2.0]), with similar results in subgroup analyses. Survival was higher with the use of pVTE prophylaxis (*p* < 0.001). We found no clear effect on CT progression (OR, 0.9; CI [0.6–1.2]). Overall, practice policies for pVTE prophylaxis vary substantially between European centers, whereas pVTE prophylaxis may contribute to improved outcome.

Trial registration number is NCT02210221 at ClinicalTrials.gov, registered on August 6, 2014 (first patient enrollment on December 19, 2014).

## Introduction

Prevention of venous thromboembolism (VTE) is less straightforward in patients with traumatic brain injury (TBI), compared to non-neurological patients admitted to the intensive care unit (ICU), because clinicians have to weigh the risks of progression of cerebral hemorrhage against the risks of VTE.^1^ Besides, compared to trauma patients, some studies suggest that patients with TBI might be at higher risk of developing VTE.^2,3^

Guidelines for patients with TBI lack high-level recommendations regarding the use of pharmaceutical VTE (pVTE) prophylaxis in patients with severe TBI, and randomized controlled trials (RCTs) on the effectiveness of pVTE prophylaxis are scarce.^4,5^ This lack of high-level evidence could result in substantial variation in pVTE prophylaxis practices. Previous studies reported wide variation in incidence rates of deep venous thrombosis (DVT) and pulmonary embolism (PE),^6^ but more recent studies suggest that the incidence rates of clinical VTE are low.^7^ When incidences of VTE are indeed low, this raises the question of whether patients with brain injuries should be given pVTE prophylaxis, especially in the acute phase during ICU care when risk of progression of intracranial hemorrhage is substantial. Previous studies have yielded conflicting results on the effectiveness and safety of pVTE prophylaxis.^8–11^ However, these studies often focus on computed tomography (CT) progression or VTE incidence alone, as opposed to long-term outcome.

The primary aim of this study is to describe the use of pVTE prophylaxis in ICU patients with TBI in European neurotrauma centers, and the secondary aim is to study the association of pVTE prophylaxis with outcome.

## Methods

### CENTER-TBI study

This study is part of the Collaborative European NeuroTrauma Effectiveness Research in Traumatic Brain Injury (CENTER-TBI) study, in which 54 ICUs from 18 countries in Europe and Israel participated.^12^ Criteria to enroll a patient in the CENTER-TBI study were 1) a clinical diagnosis of TBI, 2) indication for a head CT, and 3) presentation within 24 h after initial trauma. The single exclusion criterion was a previous history of neurological disease that could interfere with clinical outcome assessment. A more extensive description of the study and patient characteristics can be found in previous publications.^12,13^ The CENTER-TBI study included patients in three strata: emergency room (emergency department; ED), ward, and ICU. Inclusion criteria for the current analysis selected patients from the CENTER-TBI Core study who were 1) admitted to the ICU upon presentation and 2) older than 18 years. Ethics approval was obtained at each participating site. Consent for study participation was obtained according to local legislation from patient, legal representative, or next of kin, for all patients recruited.^14^

### Pharmaceutical prophylaxis

Detailed data on VTE prophylaxis were collected. Both the start and duration of pVTE prophylaxis were recorded, as well as the type of drug for pharmaceutical prophylaxis. Use of pVTE prophylaxis in this study was defined in two ways: 1) any use of pharmaceutical DVT prophylaxis at any time during the entire hospital stay and 2) use of pharmaceutical DVT prophylaxis during ICU stay.

### Outcomes

The presence of a DVT or PE during hospital admission was recorded based on clinical symptoms as per clinical practice (without routine leg ultrasound in all patients). The Glasgow Outcome Scale-Extended (GOSE) at 6 months and CT progression were the primary outcome measures. CT progression was recorded by clinicians during ICU and later hospital stay.

### Statistical analyses

Baseline characteristics are described for patients primarily admitted to the ICU with and without pVTE prophylaxis. Group differences were determined with chi-square tests for categorical variables and analysis of variance for continuous variables.

To answer the primary aim (the variation in the use of pVTE between European ICUs), a multi-variate model with pharmaceutical VTE prophylaxis as outcome and a random effect for center were used to quantify between center variation using the median odds ratio (MOR),^15^ which was further illustrated using caterpillar plots. To quantify between-country variation, an adjusted random-effects model at country level was used and illustrated in a map of Europe. The higher the random effect, the more likely a country was to use pVTE prophylaxis, even after correction for case-mix severity and random variation.

To answer the secondary aim (the effect of pVTE prophylaxis on long-term outcome), various statistical analyses were performed; at patient level, at center level, and a survival analysis. To assess the effects of pVTE prophylaxis on 6-month outcome, several analytical approaches were used at patient and center level. At patient level, an unadjusted proportional odds model was applied to assess the uncorrected relation between the use of pVTE prophylaxis and ordinal GOSE. To correct for confounding, the following variables were added: age, pupils, motor, hypotension, hypoxia, epidural hematoma (EDH), traumatic subarachnoid hemorrhage (tSAH), Marshall, Injury Severity Score (ISS), first glucose, first hemoglobin, presence of a central venous catheter, invasive blood pressure monitoring, comorbidity, American Society of Anesthesiologists (ASA), past anticoagulant use, use of tranexamic acid at the ED or ICU, cranial surgery, and extracranial surgery. We conducted multi-variate proportional odds regression analysis using these covariates, a random effect for center, and 6-month outcome. In addition, we also undertook a propensity-matched analysis, using the above covariates and pVTE prophylaxis as outcome, including center as a random effect. Patients who scored similarly on these characteristics (i.e., with similar propensity scores) were matched with the nearest neighbor method. In the matched data (selection of patients with similar characteristics), the GOSE was compared between those receiving any pharmaceutical prophylaxis and those not receiving any pharmaceutical prophylaxis. In this analysis, we additionally corrected for the covariates.

At center level, an instrumental variable (IV) analysis was performed with the percentage use of pVTE prophylaxis per center as instrument, center as random intercept, 6-month GOSE as outcome, and adjustment for the confounders as described for the patient-level analyses. We restricted this analysis to centers that contributed >10 patients to the analysis cohort. IV assumptions were checked by studying the similarity in case-mix severity of centers with lower versus more frequent use of VTE prophylaxis. Aggressive centers were defined as those using more pVTE prophylaxis than the median use, whereas non-aggressive centers were defined as those that used less pVTE prophylaxis than the median use.

The analyses described above were repeated with pVTE prophylaxis during ICU stay as an independent variable, and in several subgroups: isolated TBI patients (without major extracranial injury), patients with any traumatic intracranial lesion on CT, patients with an ICU stay of >72 h, and patients with contusions on first imaging.

Given that the effect of pharmaceutical VTE prophylaxis is also dependent on timing of treatment, we undertook a time-dependent Cox survival analysis. This Cox survival analysis was conducted with pVTE prophylaxis as a time-dependent covariate (including start and stop dates) and mortality as event. The Cox survival model with pVTE as a time-dependent covariate has two features. First, it only uses data when the patient is still “at risk” of receiving pVTE (i.e., in the hospital). Second, it takes into account the timing of pVTE, that is, patients switch from control to intervention group on the exact day they receive pVTE prophylaxis. This model was corrected using the same confounders as described above (age, pupils, motor, hypotension, hypoxia, EDH, tSAH, Marshall, ISS, first glucose, first hemoglobin, presence of a central venous catheter, invasive blood pressure monitoring, comorbidity, ASA, past anticoagulant use, and use of tranexamic acid).

A logistic regression model was used to study the effect of pVTE prophylaxis on CT progression, corrected for the confounders as described above. In addition, we studied the effect of various drugs for pVTE prophylaxis on 6-month outcome, using a multi-variate proportional odds regression, corrected for the confounders as described above. For this analysis, parnaparin, tinzaparin, heparin, and low-molecular-weight heparin (LMWH) were combined in an “other” category.

R statistical software was used for analyses. Missing data were imputed with the mice package.^16^ Data were extracted from Neurobot (version 2.1)

## Results

**FIG. 1.**
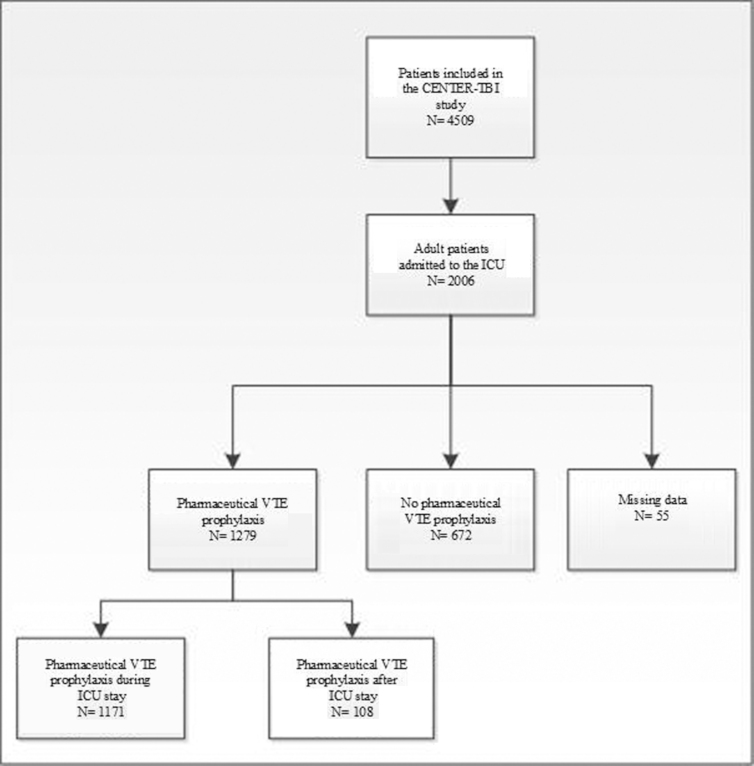
Flowchart patient inclusion in current study. Flowchart of the use of pVTE prophylaxis at ICU stay or not, including missing values. CENTER-TBI, Collaborative European NeuroTrauma Effectiveness Research in Traumatic Brain Injury; ICU, intensive care unit; VTE, venous thrombotic event.

A total of 4509 patients participated in the CENTER-TBI study. Of these, 2006 adult patients were included in the ICU stratum. The majority of these patients received pharmaceutical VTE (*N* = 1279; 64%) whereas around one third received no pVTE prophylaxis (*N* = 672; 34%). Most patients received pVTE prophylaxis during ICU stay (*N* = 1171), and in a minority of patients (*N* = 108), pVTE prophylaxis was started after ICU stay (Fig. 1).

Mechanical VTE prophylaxis was applied in around half of the cases that received pVTE prophylaxis (*N* = 657; 53%) and only in a minority with no pVTE prophylaxis (*N* = 193; 29%; Table 1).

**Table 1.** Baseline Characteristics in All ICU Admitted Patients

*N* = 1951	No pVTE prophylaxis hospital stay* N* = 672		pVTE prophylaxis hospital stay* N* = 1279		*p* value	No pVTE prophylaxis at the ICU* N* = 780		Received pVTE prophylaxis at the ICU* N* = 1171		*p* value
Age (median, IQR)	52 [33–68]		51 [33–65]		0.266	53 [34–68]		51 [32–64]		0.031
Sex, male (*N*, %)	485	(72.2)	950	(74.3)	0.343	564	(72.3)	871	(74.4)	0.335
Mechanical DVT prophylaxis	193	(28.8)	657	(53.4)	<0.001	251	(32.3)	599	(53.2)	<0.001
ISS (median, IQR)	26 [17–41]		32 [25–43]		<0.001	26 [18–38]		33 [25–43]		<0.001
GCS (*N*, %)										
Mild	270	(42.3)	382	(31.3)	<0.001	316	(42.3)	336	(30.2)	<0.001
Moderate	97	(15.2)	206	(16.9)	0.383	121	(16.2)	182	(16.4)	0.981
Severe	272	(42.6)	633	(51.8)	<0.001	310	(41.5)	595	(53.5)	<0.001
CT (*N*, %)										
tSAH	435	(73.5)	832	(75.1)	0.504	505	(73.4)	762	(75.3)	0.410
EDH	116	(19.5)	209	(18.8)	0.775	137	(19.9)	188	(18.5)	0.538
Contusion	311	(52.2)	653	(58.9)	0.009	367	(53.0)	597	(59.0)	0.017
Marshall (*N*, %)					0.514					0.315
I	64	(10.7)	117	(10.5)		74	(10.7)	107	(10.6)	
II	260	(43.6)	529	(47.7)		305	(44.1)	484	(47.7)	
III	50	(8.4)	89	(8.0)		55	(7.9)	84	(8.3)	
IV	8	(1.3)	18	(1.6)		8	(1.2)	18	(1.8)	
V/VI^a^	214	(35.9)	357	(32.2)		250	(36.1)	321	(31.7)	
Pre-injury ASA (*N*, %)					0.495					0.196
Normal healthy	363	(57.4)	682	(55.4)		409	(55.5)	636	(56.5)	
Mild systemic disease	198	(31.3)	416	(33.8)		241	(32.7)	373	(33.1)	
Severe systemic	63	(10.0)	124	(10.1)		76	(10.3)	111	(9.9)	
Severe systemic, life threat	8	(1.3)	9	(0.7)		11	(1.5)	6	(0.5)	
Cause of injury (*N*, %)					0.003					0.030
Road traffic incident	247	(38.0)	582	(47.1)		299	(39.7)	530	(46.8)	
Incidental fall	304	(46.8)	485	(39.3)		346	(45.9)	443	(39.1)	
Violence/assault	34	(5.2)	47	(3.8)		35	(4.6)	46	(4.1)	
Suicide attempt	15	(2.3)	28	(2.3)		16	(2.1)	27	(2.4)	
Other	50	(7.7)	93	(7.5)		57	(7.6)	86	(7.6)	
General VTE risk factors (*N*, %)					0.293					0.269
BMI >25	275	(55.7)	554	(52.7)		322	(55.5)	507	(52.5)	
History of VTE	4	(0.6)	15	(1.2)	0.321	6	(0.8)	13	(1.1)	0.606
Central venous catheter	207	(31.1)	586	(45.9)	<0.001	244	(31.6)	549	(46.9)	<0.001
Invasive bp monitoring	488	(72.9)	1127	(88.2)	<0.001	575	(74.0)	1040	(88.9)	<0.001
Cranial surgery	201	(30.0)	562	(44.2)	<0.001	246	(31.7)	517	(44.5)	<0.001
Extracranial surgery	105	(15.7)	451	(35.5)	<0.001	129	(16.6)	427	(36.7)	<0.001
Use of tranexamic acid	33	(4.9)	106	(8.3)	0.008	36	(4.6)	103	(8.8)	0.001
Comorbidity^b^	146	(21.7)	225	(17.6)	0.032	177	(22.7)	194	(16.6)	0.001
Length of ICU stay	2 [1–6]		10 [4–19]		<0.001	2 [1–6]		11 [4–19]		<0.001
Length of hospital stay	7 [3–14]		20 [11–36]		<0.001	8 [3–17]		21 [11–37]		<0.001
Past medication (*N*, %)					0.980					0.535
Anticoagulants	35	(5.6)	63	(5.2)		44	(6.1)	54	(4.9)	
PAI	64	(10.2)	126	(10.4)		81	(11.2)	109	(9.9)	
Both	5	(0.8)	11	(0.9)		6	(0.8)	10	(0.9)	

This table shows the baseline characteristics of TBI patients admitted to the ICU stratified by the use of pharmaceutical DVT prophylaxis (at any time during the stay).

^a^ Because a Marshall score of V rarely occurred, scores V and VI are condensed.

^b^ Cardiac (arrhythmia, valvular heart disease, congenital heart disease, thromboembolic heart disease, and ischemic heart disease), renal (renal insufficiency or failure), oncological, hepatic, or sickle cell disease.

ASA, American Society of Anesthesiologists; BMI, body mass index; bp, blood pressure; DVT, deep venous thrombosis; EDH, epidural hematoma; ICU, intensive care unit; IQR, interquartile range; ISS, Injury Severity Scale; PAI, platelet aggregation inhibitors; TBI, traumatic brain injury; tSAH, traumatic subarachnoid hemorrhage; VTE, venous thromboembolism.

### Baseline characteristics

Patients who sustained more severe injuries, based on the Glasgow Coma Scale (GCS) and ISS, were more likely to receive pVTE prophylaxis. Groups were similar regarding age and sex. A substantial proportion of patients with severe TBI received no pharmaceutical DVT prophylaxis (*N* = 272; 43%). We found no significant differences in brain injuries on CT between the pVTE and non-pVTE groups, except more contusions in the pVTE group. Factors increasing the likelihood for VTE were central venous catheter, invasive blood pressure monitoring, cranial surgery, use of tranexamic acid, and extracranial surgery. The median length of hospital stay in patients receiving pVTE prophylaxis was 20 days (interquartile range [IQR], 11–36) versus 7 days (IQR, 3–14) in patients without pVTE prophylaxis. A similar pattern was noted for patients receiving pVTE prophylaxis during ICU stay versus patients receiving no pVTE prophylaxis or after ICU stay. (Table 1) Patients treated in centers using more pVTE prophylaxis were more severely injured (based on the GCS and ISS score) and received more treatments, like invasive blood pressure monitoring, cranial and extracranial surgery, and mechanical prophylaxis (Supplementary File S1).

Overall, DVT incidence rates at the ICU (*N* = 22; 1%) and during the hospital stay (*N* = 25; 2%) were low. Further, recorded clinical PE incidence rates were low at the ICU (*N* = 20; 1%) and during the hospital stay (*N* = 24; 2%). VTE events (either DVT and/or PE) occurred in 56 patients, of whom *N* = 49 (88%) received pVTE prophylaxis and *N* = 7 (13%) did not receive pVTE prophylaxis during the hospital stay.

### Pharmaceutical prophylaxis practices

Most patients received LMWHs: enoxaparin (*N* = 517; 41%), nadroparin (*N* = 230; 18%), dalteparin (*N* = 227; 18%), tinzaparin (*N* = 48; 4%), and parnaparin (*N* = 4; 0%), whereas unfractionated heparin (*N* = 32; 3%) use was rare. The median duration of pVTE prophylaxis was 11 days (confidence interval [CI], 5–23). The median start of pVTE prophylaxis was 54.5 h after the injury (CI, 15–109; Supplementary File S2).

**FIG. 2.**
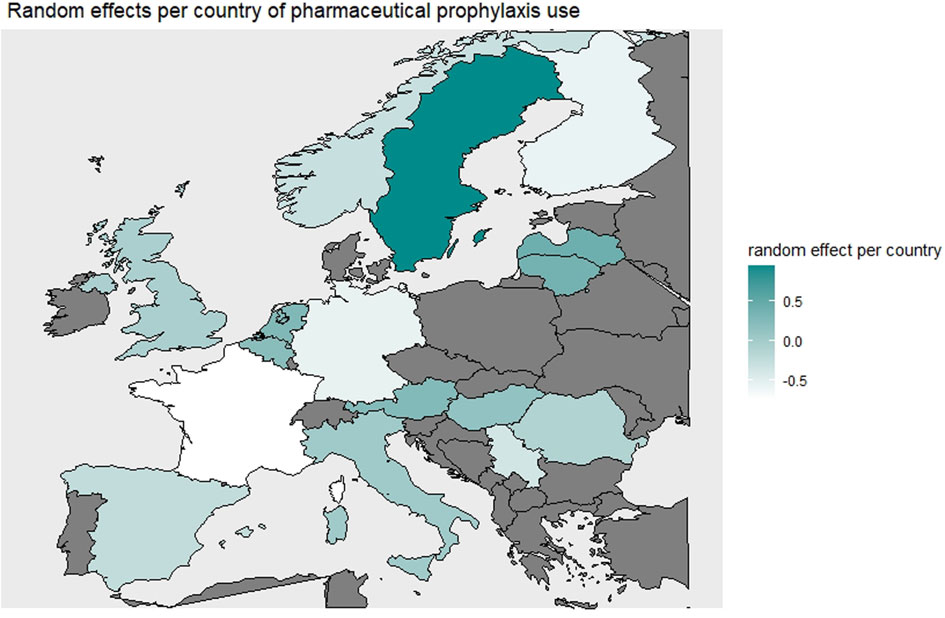
Random effects per country of pharmaceutical VTE prophylaxis use. This figure shows the variation at country level in the use of pVTE prophylaxis. This variation is corrected for case-mix severity and random variation (adjusted random effects per center). A higher random-effect estimate (darker green) represents a higher use of pVTE prophylaxis than average in that specific country, after adjustment for case-mix severity and random variation, whereas a lower random-effect estimate (white) represents a lower use of pVTE prophylaxis. pVTE, pharmaceutical VTE; VTE, venous thrombotic event.

**FIG. 3.**
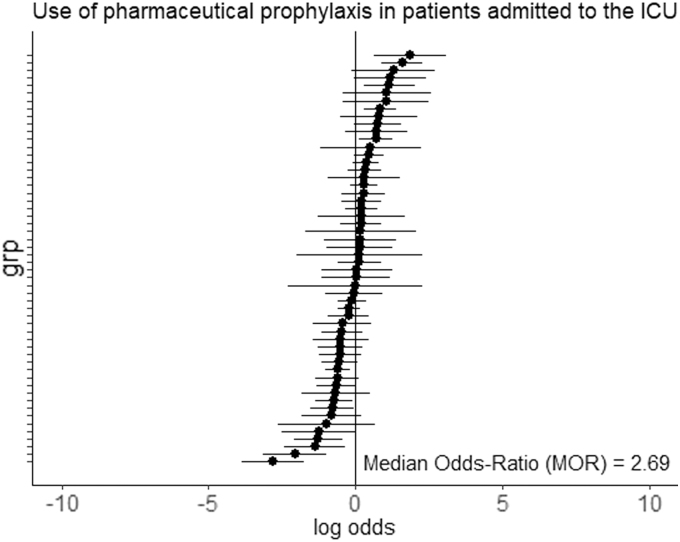
Adjusted random effects per center: use of pharmaceutical prophylaxis in patients admitted to the ICU. Variation in pVTE prophylaxis between centers in Europe. These center effects are corrected for case-mix severity per center and random variation (to show variation beyond chance). The random-effect estimates represent the use of pharmaceutical VTE prophylaxis at center level beyond case-mix severity and random variation (chance). The median odds ratio (MOR) represents the odds ratio for receiving of pharmaceutical VTE prophylaxis when comparing two randomly selected centers. An MOR of 1 indicates no differences between ICUs, whereas a larger MOR indicates higher variation between ICUs in the use of pharmaceutical VTE prophylaxis. ICU, intensive care unit; pVTE, pharmaceutical VTE; VTE, venous thrombotic event.

Overall, between-center differences in application of pVTE prophylaxis were high after case mix and random variation correction: An MOR of 2.69 was found (*p* < 0.001; Fig. 2). There was substantial variation in the application of pVTE prophylaxis between countries in Europe (Fig. 3).

### Associations with outcome

At patient level, both the adjusted multi-variate model (odds ratio [OR], 1.4 [1.1–1.7]) as well as the propensity score model (OR, 1.5 [1.1–2.0]) showed better 6-month GOSE scores in patients with pVTE prophylaxis started at some point during the hospital stay (Table 2).

**Table 2.** Associations of Pharmaceutical VTE Prophylaxis with 6-Month Outcome

	Center level		Patient level						
	IV analyses^a^		Unadjusted		Adjusted		Propensity score^b^		
Inclusion criteria	OR	[CI]	OR	[CI]	OR	[CI]	Matches	OR	[CI]
Prophylaxis during or after ICU stay									
ICU									
*N* = 2006	1.2	[0.7–2.1]	1.0	[0.8–1.2]	1.4	[1.1–1.7]	612	1.5	[1.1–2.0]
Subgroup analyses									
Isolated TBI^c^									
*N* = 900	1.0	[0.5–2.1]	0.9	[0.7–1.1]	1.2	[0.9–1.6]	340	1.3	[0.9–1.9]
Any CT lesion^d^									
*N* = 1558	1.0	[0.5–2.0]	1.1	[0.9–1.3]	1.5	[1.2–1.9]	494	1.7	[1.2–2.4]
Patients with a long ICU stay (≥72 h)									
*N* = 1315	1.1	[0.5–2.2]	1.1	[0.9–1.4]	1.6	[1.2–2.2]	237	4.3	[2.3–8.0]
Patients with contusions on imaging									
*N* = 984	1.2	[0.6–2.5]	1.2	[0.9–1.5]	1.3	[1.0–1.7]	290	1.2	[0.8–1.9]
Prophylaxis during ICU stay^*^									
ICU									
*N* = 2006	1.4	[0.8–2.5]	0.9	[0.7–1.1]	1.3	[1.0–1.6]	706	1.2	[0.9–1.5]

This table describes the association of the use of pVTE prophylaxis with GOSE at 6 months (a higher score represents a better functional outcome) among patients admitted to the ICU. The intervention is the number of patients receiving pVTE prophylaxis during or after ICU; the control group received no pVTE prophylaxis. We conducted four subgroup analyses: one with exclusion of major extracranial injuries (isolated TBI), one limited to patients with hemorrhagic CT abnormalities, one with a longer ICU stay, and one in patients with contusions on CT.

We also conducted analyses with pVTE exposure during ICU stay: ^*^Intervention group is patients who received pVTE prophylaxis at the ICU. Control group received pVTE prophylaxis after ICU stay or no pVTE prophylaxis at all.

Details of each individual analysis are as follows: At center level, an instrumental variable analyses was performed with the percentage pVTE prophylaxis as instrument, center as random intercept, corrected for case mix (extended IMPACT model and VTE risk factors), and ordinal GOSE as outcome. The analysis was restricted to centers that contributed >10 patients to the analysis. At patient level, the unadjusted model shows the relation between pharmaceutical prophylaxis use and GOSE without added confounders. The adjusted proportional odds model was corrected for case mix. A propensity-score–matched model was matched on baseline characteristics and VTE risk factors between cases (receiving pharmaceutical DVT prophylaxis) and controls (without pVTE prophylaxis). In this matched data set, the difference outcome was determined between cases and controls.

^a^ OR per 100% increase (no use of prophylaxis or use in every patient) corrected for case mix, as described above. However, for the isolated TBI patient subgroup, the ISS was not included in the analysis because of high covariance, with subgroup selection criteria.

^b^ Propensity matching used nearest neighbor with adjustment for predictors (qlogis). Analyses are pooled over different imputed data sets with different numbers of matches (number of matches is mean of matches in imputed data sets).

^c^ Exclusion major extracranial injury.

^d^ Any traumatic intracranial CT abnormality.

CI, confidence interval; CT, computed tomography; DVT, deep venous thrombosis; GOSE, Glasgow Outcome Scale-Extended; ICU, intensive care unit; IMPACT, International Mission for Prognosis and Analysis of Clinical Trials in Traumatic Brain Injury; ISS, Injury Severity Scale; IV, instrumental variable; OR, odds ratio; pVTE, prophylaxis: pharmaceutical VTE prophylaxis; TBI, traumatic brain injury; VTE, venous thrombosis events.

At center level, no major differences in patient population (case mix) between aggressive and non-aggressive centers were found for patient characteristics regarding injury severity (Supplementary File 2). A comparable association between pVTE prophylaxis and outcome was found, but this did not reach significance (OR, 1.2 [0.7–2.1]; Table 2).

Analysis of pVTE prophylaxis in the subgroup analyses at patient level did show associations with improved outcome, although this did not reach statistical significance in all subgroups. Effect estimates for the use of pVTE prophylaxis during ICU stay were similar as well (Table 2).

**FIG. 4.**
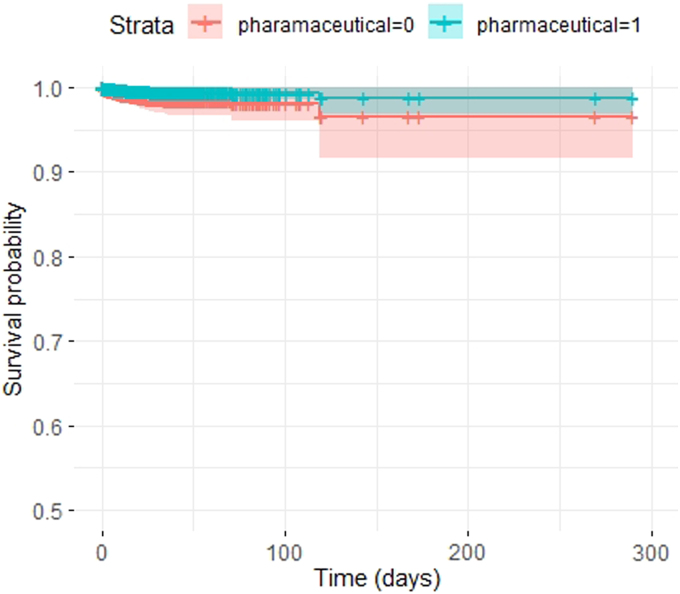
Time-dependent Cox survival curve. This figure shows the time-dependent Cox survival curve for the use of pVTE prophylaxis. The difference in survival is significant (*p* < 0.001), favoring the use of VTE prophylaxis. The y-axis of survival starts at 0.5. Time is in days. pVTE, pharmaceutical VTE; VTE, venous thrombotic event.

Survival analyses also showed a beneficial effect of pVTE prophylaxis on survival (*p* < 0.001; Fig. 4). No effect on clinical CT progression was found (OR, 0.9; CI, 0.6–1.2). When analyzing the effect of different drugs for pVTE prophylaxis, we found that they all were associated with improved outcome compared to no pVTE prophylaxis, all with a comparable effect size: enoxaparin (OR, 1.5 [1.2–1.9]); dalteparin (OR, 1.4 [1.0–1.9]); nadroparin (OR, 1.2 [0.9–1.7]); and other (OR, 1.5 [1.2–2.0]).

## Discussion

Substantial variation at country and center level was found in the use of pVTE prophylaxis, beyond case-mix differences and random variation. Use of pVTE prophylaxis was associated with improved outcome after 6 months, when administered at the ICU or subsequently at the ward. Overall, this indicates that VTE prophylaxis seems safe and might improve outcomes in critically ill TBI patients. However, given the low incidence of clinically evident VTEs, the pathophysiology explaining this association is not clear from this analysis.

To our knowledge, no previous studies have described between-center variation in pVTE prophylaxis. These variations were observed at both center and country level, and persisted despite correction for random variation and case-mix, suggesting that application of pVTE prophylaxis is driven by hospital policy and local clinical culture. We found a low reported incidence of VTE in patients with TBI in neurotrauma centers in Europe. Although higher incidences of VTE have been reported in previous studies in TBI,^6,17^ others reported similar or even lower percentages compared with our study.^5,7^ The discrepancy between higher incidences in other studies may be partly explained either by a lack of routine lower-limb screening with ultrasound for DVT or clinical under-reporting of DVT in our study. In our study, no conclusion on these outcomes (DVT and PE) could be drawn given that the statistical power was very low.

The association between pVTE prophylaxis and potentially improved functional outcome after 6 months suggests that the benefits of pVTE prophylaxis may outweigh the risks. This result was consistent among all level analyses performed, strengthening the finding of the direction of the effect (improved outcome) and rendering the possibility of a harmful effect less likely. In addition, patients receiving pVTE were more severely injured (based on the GCS and ISS score, number of contusions on CT, and length of stay) compared with patients without pVTE prohylaxis, indicating that in the case of insufficient adjustment, the association between the use of pVTE prophylaxis and improved outcome would be even stronger. Also, no effect of pVTE prophylaxis on CT expansion was found. Previous large studies on the effectiveness of pVTE prophylaxis did not translate to high-level evidence.^18^ At center level, the analyses did not reach significance, but interpretation of these results is difficult given that the statistical power at center level was very low.^19^ Similar associations with outcome after 6 months were found with the use of pVTE prophylaxis during ICU stay. Survival analyses also showed an improvement in survival during the hospital stay. Overall, our results suggest that providing pVTE prophylaxis might improve outcome.

The mechanisms behind possibly improved outcome might be less straightforward than currently thought, given the low incidence of clinical VTE. When taken at face value, the outcome associations found may appear to be less likely caused by a decrease of VTE in patients treated with pVTE prophylaxis (given very low incidence in our study) and may therefore indicate a protective effect of this treatment attributable to mechanisms not yet elucidated. One hypothesis might be that pVTE prophylaxis might reduce microthrombi in the penumbra of contusional lesions.^20–22^ The hypothesis was substantiated by a beneficial result of pVTE prophylaxis in the subgroup analyses with patients with contusions. Another hypothesis is that the use of pVTE prophylaxis might improve outcome beyond that expected by the antithrombotic effect, which might be attributable to an anti-inflammatory effect of pVTE prophylaxis and might reduce neuroinflammation. This beneficial anti-inflammatory effect of pVTE prophylaxis is already shown in various mouse models, but needs to be confirmed in future trials.^23,24^ Others might argue that patients without pVTE prophylaxis would receive mechanical VTE prophylaxis instead. However, this was not confirmed in our results (only around one third of patients without pVTE prophylaxis received mechanical prophylaxis instead).

This study has several strengths and limitations. In the CENTER-TBI study, multiple neurotrauma centers participated from different countries, enabling us to study between-center variation and effectiveness at center level. Several statistical methods were applied. These methods complement each other in their advantages and disadvantages.^19^ For example, center-level analyses (instrumental variable analyses) are suitable to abolish effects of unmeasured confounding, whereas the power of patient-level analyses is higher, in spite of only being able to adjust for measured confounders. Further, we performed survival analysis to correct for the substantial difference in length of stay between patients with and without pVTE.

This study also has its limitations. CT progression during hospital stay was scored by clinicians subjectively without accounting for a time component. Further routine CT follow-up was not prescribed at specific time points in the protocol. So, it could be that pVTE prophylaxis was administrated after the CT progression occurred. Also, CT progression was not clearly defined (e.g., only progression of cerebral bleeding or other traumatic lesions). The longer length of ICU and hospital stay in patients receiving pVTE prophylaxis compared to the non-pVTE group suggest the possibility of different subpopulations (and a potential higher risk profile in patients receiving pVTE). However, although residual confounding cannot be excluded, the IV analyses and different statistical approaches should account for residual confounding and show similar directions of the effect.

Future studies are needed to elucidate the mechanism behind the beneficial effect of pVTE prophylaxis and determine the best time to initiate prophylaxis. An additional quantitative volumetric analysis of CT progression would be sensitive. In the ideal scenario, an RCT should be considered to confirm our findings, which were obtained in an observational study utilizing comparative effectiveness approaches. However, the extreme heterogeneity of the TBI population in the ICU may render a strict protocol with standardization on when to apply the pVTE prophylaxis challenging.

## Conclusion

Substantial between-center variation exists in the use of pVTE prophylaxis, whereas pVTE prophylaxis might be associated with improved 6-month functional outcome and lower mortality rates, without CT progression. Therefore, although VTE prophylaxis is likely to be safe, further research should be conducted to confirm and elucidate the associations and should be aimed at a better selection of patients more likely to benefit from this treatment.

### Availability of data and materials

The used data sets that are analyzed in this study are available after a reasonable request.^14^

### Ethics approval

In each recruiting center, ethical approval was given; an online overview is available (https://www.center-tbi.eu/project/ethical-approval). Consent for study participation was obtained according to local legislation from the patient, legal representative, or next of kin, for all patients recruited.^14^

## Supplementary Material

Supplemental data

Supplemental data

Supplemental data

## Supplementary Material

Supplementary File S2 (figure)

Supplementary File S3 (collaborator's list)

## Abbreviations Used

ASA: American Society of Anesthesiologists
CI: confidence interval
CT: computed tomography
DVT: deep venous thrombosis
ED: emergency department
EDH: epidural hematoma
GCS: Glasgow Coma Scale
GOSE: Glasgow Outcome Scale-Extended
ICU: intensive care unit
IQR: interquartile range
ISS: Injury Severity Score
IV: instrumental variable
LMWH: low-molecular-weight heparin
OR: odds ratio
PE: pulmonary embolism
pVTE: pharmaceutical VTE
RCTs: randomized controlled trials
TBI: traumatic brain injury
tSAH: traumatic subarachnoid hemorrhage
VTE: venous thromboembolism
