# Supplementary material for: Pharmaceutical Venous Thrombosis Prophylaxis in Critically Ill Traumatic Brain Injury Patients
Source: Neurotrauma Rep. 2022 Jan 7;2(1):4–14. doi: 10.1089/neur.2021.0037 (PMC8804253; doi:10.1089/neur.2021.0037)
Supplement: Supplemental data [file Supp_FileS3.docx]

**The CENTER-TBI ICU WP6 participants and ICU ONLY investigators:**

Cecilia Åkerlund^1^, Krisztina Amrein ^2^, Nada Andelic^3^, Lasse Andreassen^4^, Audny Anke^5^, Gérard Audibert^6^, Philippe Azouvi^7^, Maria Luisa Azzolini^8^, Ronald Bartels^9^, Ronny Beer^10^, Bo‑Michael Bellander^11^, Habib Benali^12^, Maurizio Berardino^13^, Luigi Beretta^8^, Erta Beqiri^14^, Morten Blaabjerg^15^, Stine Borgen Lund^16^, Camilla Brorsson^17^, Andras Buki^18^, Manuel Cabeleira^19^, Alessio Caccioppola^20^, Emiliana Calappi^20^, Maria Rosa Calvi^8^, Peter Cameron^21^, Guillermo Carbayo Lozano^22^, Marco Carbonara^20^, Ana M. Castaño‑León^23^, Simona Cavallo^13^, Giorgio Chevallard^14^, Arturo Chieregato^14^, Giuseppe Citerio^24, 25^, Hans Clusmann^26^, Mark Coburn^27^, Jonathan Coles^28^, Jamie D. Cooper^29^, Marta Correia^30^, Endre Czeiter^18^, Marek Czosnyka^19^, Claire Dahyot‑Fizelier^31^, Paul Dark^32^, Véronique De Keyser^33^, Vincent Degos^12^, Francesco Della Corte^34^, Hugo den Boogert^9^, Bart Depreitere^35^, Dula Dilvesi^36^, Abhishek Dixit^37^, Jens Dreier^38^, Guy‑Loup Dulière^39^, Ari Ercole^37^, Erzsébet Ezer^40^, Martin Fabricius^41^, Kelly Foks^42^, Shirin Frisvold^43^, Alex Furmanov^44^, Damien Galanaud^12^, Dashiell Gantner^21^, Alexandre Ghuysen^45^, Lelde Giga^46^, Jagos Golubovic^36^, Pedro A. Gomez^23^, Benjamin Gravesteijn^47^, Francesca Grossi^34^, Deepak Gupta^48^, Iain Haitsma^49^, Raimund Helbok^10^, Eirik Helseth^50^, Jilske Huijben^47^, Peter J. Hutchinson^51^, Stefan Jankowski^52^, Faye Johnson^53^, Mladen Karan^36^, Angelos G. Kolias^51^, Daniel Kondziella^41^, Evgenios Kornaropoulos^37^, Lars‑Owe Koskinen^54^, Noémi Kovács^55^, Ana Kowark^56^, Alfonso Lagares^23^, Steven Laureys^57^, Fiona Lecky^58,59^, Didier Ledoux^57^, Aurelie Lejeune^60^, Roger Lightfoot^61^, Hester Lingsma^47^, Andrew I.R. Maas^33^, Alex Manara^62^, Costanza Martino^63^, Hugues Maréchal^39^, Julia Mattern^64^, Catherine McMahon^65^, David Menon^37^, Tomas Menovsky^33^, Benoit Misset^57^, Visakh Muraleedharan^66^, Lynnette Murray^21^, Ancuta Negru^67^, David Nelson^1^, Virginia Newcombe^37^, József Nyirádi^2^, Fabrizio Ortolano^20^, Jean‑François Payen^68^, Vincent Perlbarg^12^, Paolo Persona^69^, Wilco Peul^70^, Anna Piippo-Karjalainen^71^, Horia Ples^67^, Inigo Pomposo^22^, Jussi P. Posti^72^, Louis Puybasset^73^, Andreea Radoi^74^, Arminas Ragauskas^75^, Rahul Raj^71^, Jonathan Rhodes^76^, Sophie Richter^37^, Saulius Rocka^75^, Cecilie Roe^77^, Olav Roise^78,79^, Jeffrey V. Rosenfeld^80^, Christina Rosenlund^81^, Guy Rosenthal^44^, Rolf Rossaint^56^, Sandra Rossi^69^, Juan Sahuquillo^74^, Oddrun Sandrød ^82^, Oliver Sakowitz^64, 83^, Renan Sanchez‑Porras^83^, Charlie Sewalt^47^, Kari Schirmer-Mikalsen^82, 84^, Rico Frederik Schou^85^, Peter Smielewski^19^, Abayomi Sorinola^86^, Emmanuel Stamatakis^37^, Ewout W. Steyerberg^47,^ ^87^, Nino Stocchetti^88^, Nina Sundström^89^, Riikka Takala^90^, Viktória Tamás^86^, Tomas Tamosuitis^91^, Olli Tenovuo^72^, Matt Thomas^62^, Dick Tibboel^92^, Christos Tolias^93^, Tony Trapani^21^, Cristina Maria Tudora^67^, Andreas Unterberg^64^, Peter Vajkoczy^94^, Shirley Vallance^21^, Egils Valeinis ^46^, Zoltán Vámos^40^, Mathieu van der Jagt^95^, Gregory Van der Steen^33^, Jeroen T.J.M. van Dijck^70^, Thomas A. van Essen^70^, Roel P. J. van Wijk^70^, Alessia Vargiolu^25^, Emmanuel Vega^60^, Anne Vik^84, 96^, Rimantas Vilcinis^91^, Victor Volovici^49^, Daphne Voormolen^47^, Petar Vulekovic^36^, Eveline Wiegers^47^, Guy Williams^37^, Stefan Winzeck^37^, Stefan Wolf^97^, Alexander Younsi^64^, Frederick A. Zeiler^37,98^, Agate Ziverte^46^ , Tommaso Zoerle^20^

^1^ Department of Physiology and Pharmacology, Section of Perioperative Medicine and Intensive Care, Karolinska Institutet, Stockholm, Sweden

^2^ János Szentágothai Research Centre, University of Pécs, Pécs, Hungary

^3^ Division of Surgery and Clinical Neuroscience, Department of Physical Medicine and Rehabilitation, Oslo University Hospital and University of Oslo, Oslo, Norway

^4^ Department of Neurosurgery, University Hospital Northern Norway, Tromso, Norway

^5^ Department of Physical Medicine and Rehabilitation, University Hospital Northern Norway, Tromso, Norway

^6^ Department of Anesthesiology & Intensive Care, University Hospital Nancy, Nancy, France

^7^ Raymond Poincare hospital, Assistance Publique – Hopitaux de Paris, Paris, France

^8^ Department of Anesthesiology & Intensive Care, S Raffaele University Hospital, Milan, Italy

^9^ Department of Neurosurgery, Radboud University Medical Center, Nijmegen, The Netherlands

^10^ Department of Neurology, Neurological Intensive Care Unit, Medical University of Innsbruck, Innsbruck, Austria

^11^ Department of Neurosurgery & Anesthesia & intensive care medicine, Karolinska University Hospital, Stockholm, Sweden

^12^ Anesthesie-Réanimation, Assistance Publique – Hopitaux de Paris, Paris, France

^13^ Department of Anesthesia & ICU, AOU Città della Salute e della Scienza di Torino - Orthopedic and Trauma Center, Torino, Italy

^14^ NeuroIntensive Care, Niguarda Hospital, Milan, Italy

^15^ Department of Neurology, Odense University Hospital, Odense, Denmark

^16^ Department of Public Health and Nursing, Faculty of Medicine and health Sciences, Norwegian University of Science and Technology, NTNU, Trondheim, Norway

**^17^**Department of Surgery and Perioperative Science, **Umeå University, Umeå, Sweden**

^18^ Department of Neurosurgery, Medical School, University of Pécs, Hungary and Neurotrauma Research Group, János Szentágothai Research Centre, University of Pécs, Hungary

^19^ Brain Physics Lab, Division of Neurosurgery, Dept of Clinical Neurosciences, University of Cambridge, Addenbrooke’s Hospital, Cambridge, UK

^20^ Neuro ICU, Fondazione IRCCS Cà Granda Ospedale Maggiore Policlinico, Milan, Italy

^21^ ANZIC Research Centre, Monash University, Department of Epidemiology and Preventive Medicine, Melbourne, Victoria, Australia

^22^ Department of Neurosurgery, Hospital of Cruces, Bilbao, Spain

^23^ Department of Neurosurgery, Hospital Universitario 12 de Octubre, Madrid, Spain

^24^ School of Medicine and Surgery, Università Milano Bicocca, Milano, Italy

^25^ NeuroIntensive Care, ASST di Monza, Monza, Italy

^26^ Department of Neurosurgery, Medical Faculty RWTH Aachen University, Aachen, Germany

^27^ Department of Anesthesiology and Intensive Care Medicine, University Hospital Bonn, Bonn, Germany

^28^ Department of Anesthesia & Neurointensive Care, Cambridge University Hospital NHS Foundation Trust, Cambridge, UK

^29^ School of Public Health & PM, Monash University and The Alfred Hospital, Melbourne, Victoria, Australia

^30^ Radiology/MRI department, MRC Cognition and Brain Sciences Unit, Cambridge, UK

^31^ Intensive Care Unit, CHU Poitiers, Potiers, France

^32^ University of Manchester NIHR Biomedical Research Centre, Critical Care Directorate,  Salford Royal Hospital NHS Foundation Trust, Salford, UK.

^33^ Department of Neurosurgery, Antwerp University Hospital and University of Antwerp, Edegem, Belgium

^34^ Department of Anesthesia & Intensive Care, Maggiore Della Carità Hospital, Novara, Italy

^35^ Department of Neurosurgery, University Hospitals Leuven, Leuven, Belgium

^36^ Department of Neurosurgery, Clinical centre of Vojvodina, Faculty of Medicine, University of Novi Sad, Novi Sad, Serbia

^37^ Division of Anaesthesia, University of Cambridge, Addenbrooke’s Hospital, Cambridge, UK

^38^ Center for Stroke Research Berlin, Charité – Universitätsmedizin Berlin, corporate member of Freie Universität Berlin, Humboldt-Universität zu Berlin, and Berlin Institute of Health, Berlin, Germany

^39^ Intensive Care Unit, CHR Citadelle, Liège, Belgium

^40^ Department of Anaesthesiology and Intensive Therapy, University of Pécs, Pécs, Hungary

^41^ Departments of Neurology, Clinical Neurophysiology and Neuroanesthesiology, Region Hovedstaden Rigshospitalet, Copenhagen, Denmark

^42^ Department of Neurology, Erasmus MC, Rotterdam, the Netherlands

^43^ Department of Anesthesiology and Intensive care, University Hospital Northern Norway, Tromso, Norway

^44^ Department of Neurosurgery, Hadassah-hebrew University Medical center, Jerusalem, Israel

^45^ Emergency Department, CHU, Liège, Belgium

^46^ Neurosurgery clinic, Pauls Stradins Clinical University Hospital, Riga, Latvia

^47^ Department of Public Health, Erasmus Medical Center-University Medical Center, Rotterdam, The Netherlands

^48^ Department of Neurosurgery, Neurosciences Centre & JPN Apex trauma centre, All India Institute of Medical Sciences, New Delhi-110029, India

^49^ Department of Neurosurgery, Erasmus MC, Rotterdam, the Netherlands

^50^ Department of Neurosurgery, Oslo University Hospital, Oslo, Norway

^51^ Division of Neurosurgery, Department of Clinical Neurosciences, Addenbrooke’s Hospital & University of Cambridge, Cambridge, UK

^52^ Neurointensive Care , Sheffield Teaching Hospitals NHS Foundation Trust, Sheffield, UK

^53^ Salford Royal Hospital NHS Foundation Trust Acute Research Delivery Team, Salford, UK

**^54^**Department of Clinical Neuroscience, Neurosurgery, **Umeå University, Umeå, Sweden**

^55^ Hungarian Brain Research Program - Grant No. KTIA_13_NAP-A-II/8, University of Pécs, Pécs, Hungary

^56^ Department of Anaesthesiology, University Hospital of Aachen, Aachen, Germany

^57^ Cyclotron Research Center , University of Liège, Liège, Belgium

^58^ Centre for Urgent and Emergency Care Research (CURE), Health Services Research Section, School of Health and Related Research (ScHARR), University of Sheffield, Sheffield, UK

^59^ Emergency Department, Salford Royal Hospital, Salford UK

^60^ Department of Anesthesiology-Intensive Care, Lille University Hospital, Lille, France

^61^ Department of Anesthesiology & Intensive Care, University Hospitals Southhampton NHS Trust, Southhampton, UK

^62^ Intensive Care Unit, Southmead Hospital, Bristol, Bristol, UK

^63^ Department of Anesthesia & Intensive Care,M. Bufalini Hospital, Cesena, Italy

^64^ Department of Neurosurgery, University Hospital Heidelberg, Heidelberg, Germany

^65^ Department of Neurosurgery, The Walton centre NHS Foundation Trust, Liverpool, UK

^66^ Karolinska Institutet, INCF International Neuroinformatics Coordinating Facility, Stockholm, Sweden

^67^ Department of Neurosurgery, Emergency County Hospital Timisoara , Timisoara, Romania

^68^ Department of Anesthesiology & Intensive Care, University Hospital of Grenoble, Grenoble, France

^69^ Department of Anesthesia & Intensive Care, Azienda Ospedaliera Università di Padova, Padova, Italy

^70^ Dept. of Neurosurgery, Leiden University Medical Center, Leiden, The Netherlands and Dept. of Neurosurgery, Medical Center Haaglanden, The Hague, The Netherlands

^71^ Department of Neurosurgery, Helsinki University Central Hospital

^72^ Division of Clinical Neurosciences, Department of Neurosurgery and Turku Brain Injury Centre, Turku University Hospital and University of Turku, Turku, Finland

^73^ Department of Anesthesiology and Critical Care, Pitié -Salpêtrière Teaching Hospital, Assistance Publique, Hôpitaux de Paris and University Pierre et Marie Curie, Paris, France

^74^ Neurotraumatology and Neurosurgery Research Unit (UNINN), Vall d'Hebron Research Institute, Barcelona, Spain

^75^ Department of Neurosurgery, Kaunas University of technology and Vilnius University, Vilnius, Lithuania

^76^ Department of Anaesthesia, Critical Care & Pain Medicine NHS Lothian & University of Edinburg, Edinburgh, UK

^77^ Department of Physical Medicine and Rehabilitation, Oslo University Hospital/University of Oslo, Oslo, Norway

^78^ Division of Orthopedics, Oslo University Hospital, Oslo, Norway

^79^ Institute of Clinical Medicine, Faculty of Medicine, University of Olso, Oslo, Norway

^80^ National Trauma Research Institute, The Alfred Hospital, Monash University, Melbourne, Victoria, Australia

^81^ Department of Neurosurgery, Odense University Hospital, Odense, Denmark

^82^ Department of Anasthesiology and Intensive Care Medicine, St.Olavs Hospital, Trondheim University Hospital, Trondheim, Norway

^83^ Klinik für Neurochirurgie, Klinikum Ludwigsburg, Ludwigsburg, Germany

^84^ Department of Neuromedicine and Movement Science, Norwegian University of Science and Technology, NTNU, Trondheim, Norway

^85^ Department of Neuroanesthesia and Neurointensive Care, Odense University Hospital, Odense, Denmark

^86^ Department of Neurosurgery, University of Pécs, Pécs, Hungary

^87^ Dept. of Department of Biomedical Data Sciences, Leiden University Medical Center, Leiden, The Netherlands

^88^ Department of Pathophysiology and Transplantation, Milan University, and Neuroscience ICU, Fondazione IRCCS Cà Granda Ospedale Maggiore Policlinico, Milano, Italy

**^89^**Department of Radiation Sciences, Biomedical Engineering, **Umeå University, Umeå, Sweden**

^90^ Perioperative Services, Intensive Care Medicine and Pain Management, Turku University Hospital and University of Turku, Turku, Finland

^91^ Department of Neurosurgery, Kaunas University of Health Sciences, Kaunas, Lithuania

^92^ Intensive Care and Department of Pediatric Surgery, Erasmus Medical Center, Sophia Children’s Hospital, Rotterdam, The Netherlands

^93^ Department of Neurosurgery, Kings college London, London, UK

^94^ Neurologie, Neurochirurgie und Psychiatrie, Charité – Universitätsmedizin Berlin, Berlin, Germany

^95^ Department of Intensive Care Adults, Erasmus MC– University Medical Center Rotterdam, Rotterdam, the Netherlands

^96^ Department of Neurosurgery, St.Olavs Hospital, Trondheim University Hospital, Trondheim, Norway

^97^ Department of Neurosurgery, Charité – Universitätsmedizin Berlin, corporate member of Freie Universität Berlin, Humboldt-Universität zu Berlin, and Berlin Institute of Health, Berlin, Germany

^98^ Section of Neurosurgery, Department of Surgery, Rady Faculty of Health Sciences, University of Manitoba, Winnipeg, MB, Canada

| Cecilia | Ackerlund | cecilia.ai.akerlund@gmail.com |
| --- | --- | --- |
| Krisztina | Amrein | tina.amrein84@gmail.com |
| Nada | Andelic | NADAND@ous-hf.no |
| Lasse | Andreassen | Lasse.Andreassen@unn.no |
| Audny | Anke | [Audny.anke@unn.no](mailto:Audny.anke@unn.no) |
| Gérard | Audibert | g.audibert@chu-nancy.fr |
| Philippe | Azouvi | philippe.azouvi@rpc.aphp.fr |
| Maria Luisa | Azzolini | [azzolini.marialuisa@hsr.it](mailto:azzolini.marialuisa@hsr.it) |
| Ronald | Bartels | Ronald.Bartels@radboudumc.nl |
| Ronny | Beer | ronny.beer@i-med.ac.at |
| Bo-Michael | Bellander | bo-michael.bellander@karolinska.se |
| Habib | Benali | habib.benali@gmail.com |
| Maurizio | Berardino | maurizio_berardino@fastwebnet.it |
| Luigi | Beretta | beretta.luigi@hsr.it |
| Erta | Beqiri | erta.beqiri@gmail.com |
| Morten | Blaabjerg | [morten.blaabjerg1@rsyd.dk](mailto:morten.blaabjerg1@rsyd.dk) |
| Stine | Borgen Lund | stine.b.lund@ntnu.no |
| Camilla | Brorsson | [Camilla.Brorsson@umu.se](mailto:Camilla.Brorsson@umu.se) |
| Andras | Buki | 2saturn@gmail.com |
| Manuel | Cabeleira | mc916@cam.ac.uk |
| Alessio | Caccioppola | alessio.caccioppola@gmail.com |
| Emiliana | Calappi | [calemy02@yahoo.it](mailto:calemy02@yahoo.it) |
| Maria Rosa | Calvi | [calvi.mariarosa@hsr.it](mailto:calvi.mariarosa@hsr.it) |
| Peter | Cameron | [peter.cameron@med.monash.edu.au](mailto:peter.cameron@med.monash.edu.au) |
| Guillermo | Carbayo Lozano | guillermobilbo@gmail.com |
| Marco | Carbonara | marco.carbonara@gmail.com |
| Ana M. | Castaño-León | [ana.maria.castano.leon@gmail.com](mailto:ana.maria.castano.leon@gmail.com) |
| Simona | Cavallo | cavallosimona1@gmail.com |
| Giorgio | Chevallard | [giorgio.chevallard@ospedaleniguarda.it](mailto:giorgio.chevallard@ospedaleniguarda.it) |
| Arturo | Chieregato | [arturo.chieregato@ospedaleniguarda.it](mailto:arturo.chieregato@ospedaleniguarda.it) |
| Giuseppe | Citerio | giuseppe.citerio@unimib.it |
| Hans | Clusmann | hclusmann@ukaachen.be |
| Mark Steven | Coburn | mark.coburn@ukbonn.de |
| Jonathan | Coles | jpc44@wbic.cam.ac.uk |
| Jamie D. | Cooper | jamie.cooper@monash.edu |
| Marta | Correia | Marta.Correia@mrc-cbu.cam.ac.uk |
| Endre | Czeiter | endre.czeiter@gmail.com |
| Marek | Czosnyka | mc141@medschl.cam.ac.uk |
| Claire | Dahyot-Fizelier | c.dahyot-fizelier@chu-poitiers.fr |
| Paul | Dark | paul.m.dark@manchester.ac.uk |
| Véronique | De Keyser | [veronique.dekeyser@uza.be](mailto:veronique.dekeyser@uza.be) |
| Vincent | Degos | [vincent.degos@aphp.fr](mailto:vincent.degos@aphp.fr) |
| Francesco | Della Corte | dellacorte.f@gmail.com |
| Hugo | den Boogert | Hugo.denBoogert@radboudumc.nl |
| Bart | Depreitere | bart.depreitere@uzleuven.be |
| Đula | Đilvesi | [djuladjilvesi@gmail.com](mailto:djuladjilvesi@gmail.com) |
| Abhishek | Dixit | ad825@cam.ac.uk |
| Jens | Dreier | jens.dreier@charite.de |
| Guy-Loup | Dulière | glduliere@gmail.com |
| Ari | Ercole | ae105@cam.ac.uk |
| Erzsébet | Ezer | ezererzsebet@yahoo.com |
| Martin | Fabricius | fabricius@dadlnet.dk |
| Kelly | Foks | k.foks@erasmusmc.nl |
| Shirin | Frisvold | Shirin.Kordasti@unn.no |
| Alex | Furmanov | alexpuil@yahoo.com |
| Damien | Galanaud | galanaud@gmail.com |
| Dashiell | Gantner | dashiell.gantner@monash.edu |
| Alexandre | Ghuysen | [A.Ghuysen@chu.ulg.ac.be](mailto:A.Ghuysen@chu.ulg.ac.be) |
| Lelde | Giga | lelde.giga@inbox.lv |
| Jagoš | Golubović | [jagosgolubovic@gmail.com](mailto:jagosgolubovic@gmail.com) |
| Pedro A. | Gomez | [pagolopez@gmail.com](mailto:pagolopez@gmail.com) |
| Benjamin | Gravesteijn | b.gravesteijn@erasmusmc.nl |
| Francesca | Grossi | francesca.grossi@libero.it |
| Deepak | Gupta | drdeepakgupta@gmail.com |
| Iain | Haitsma | i.haitsma@erasmusmc.nl |
| Raimund | Helbok | Raimund.Helbok@tirol-kliniken.at |
| Eirik | Helseth | EHELSETH@ous-hf.no |
| Jilske | Huijben | j.a.huijben@erasmusmc.nl |
| Peter J. | Hutchinson | pjah2@cam.ac.uk |
| Stefan | Jankowski | Stefan.Jankowski@sth.nhs.uk |
| Faye | Johnson | faye.johnson@live.co.uk |
| Mladen | Karan | mladjokaran@gmail.com |
| Angelos G. | Kolias | angeloskolias@gmail.com |
| Daniel | Kondziella | Daniel.Kondziella@regionh.dk |
| Evgenios | Kornaropoulos | ek481@cam.ac.uk |
| Lars-Owe | Koskinen | [Lars-Owe.Koskinen@umu.se](mailto:Lars-Owe.Koskinen@umu.se) |
| Noémi | Kovács | kovacs.noemi@pte.hu |
| Ana | Kowark | akowark@ukaachen.de |
| Alfonso | Lagares | algadoc@yahoo.com |
| Steven | Laureys | [steven.laureys@ulg.ac.be](mailto:steven.laureys@ulg.ac.be) |
| Fiona | Lecky | f.e.lecky@sheffield.ac.uk |
| Didier | Ledoux | dledoux@chu.ulg.ac.be |
| Roger | Lightfoot | Roger.Lightfoot@uhs.nhs.uk |
| Hester | Lingsma | h.lingsma@erasmusmc.nl |
| Andrew I.R. | Maas | [andrew.maas@uza.be](mailto:andrew.maas@uza.be) |
| Alex | Manara | Alex.Manara@nbt.nhs.uk |
| Hugues | Maréchal | Hugues.Marechal@chrcitadelle.be |
| Costanza | Martino | costmartino74@gmail.com |
| Julia | Mattern | Julia.Mattern@med.uni-heidelberg.de |
| Catherine | McMahon | Catherine.McMahon@thewaltoncentre.nhs.uk |
| David | Menon | dkm13@cam.ac.uk |
| Tomas | Menovsky | [tomas.menovsky@uza.be](mailto:tomas.menovsky@uza.be) |
| Benoit | Misset | Benoit.Misset@chuliege.be |
| Visakh | Muraleedharan | visakh@incf.org |
| Lynnette | Murray | lynnette.murray@monash.edu |
| Ancuta | Negru | [negruancu@gmail.com](mailto:negruancu@gmail.com) |
| David | Nelson | david.nelson@karolinska.se |
| Virginia | Newcombe | vfjn2@cam.ac.uk |
| József | Nyirádi | nyiradi.jozsef@pte.hu |
| Fabrizio | Ortolano | [lupeda@gmail.com](mailto:lupeda@gmail.com) |
| Jean-François | Payen | Jean-Francois.Payen@ujf-grenoble.fr |
| Vincent | Perlbarg | vincent.perlbarg@gmail.com |
| Paolo | Persona | ppersona75@gmail.com |
| Wilco | Peul | W.C.Peul@lumc.nl |
| Anna | Piippo-Karjalainen | anna.piippo@hus.fi |
| Horia | Ples | horia.ples@neuromed.ro |
| Inigo | Pomposo | inigo.pomposo@osakidetza.net |
| Jussi P. | Posti | [jussi.posti@tyks.fi](mailto:jussi.posti@tyks.fi) |
| Louis | Puybasset | louis.puybasset@aphp.fr |
| Andreea | Rădoi | [aradoi@neurotrauma.net](mailto:aradoi@neurotrauma.net) |
| Arminas | Ragauskas | telematics@ktu.lt |
| Rahul | Raj | [rahul.raj@hus.fi](mailto:rahul.raj@hus.fi) |
| Jonathan | Rhodes | jrhodes1@staffmail.ed.ac.uk |
| Sophie | Richter | sr773@cam.ac.uk |
| Saulius | Rocka | saulius.rocka@mf.vu.lt |
| Cecilie | Roe | e.c.t.roe@medisin.uio.no |
| Olav | Roise | olav.roise@medisin.uio.no |
| Jeffrey | Rosenfeld | J.Rosenfeld@alfred.org.au |
| Christina | Rosenlund | chrisstenrose@gmail.com |
| Guy | Rosenthal | [rosenthalg@hadassah.org.il](mailto:rosenthalg@hadassah.org.il) |
| Rolf | Rossaint | RRossaint@ukaachen.de |
| Sandra | Rossi | sandrarossi0@gmail.com |
| Juan | Sahuquillo | sahuquillo@neurotrauma.net |
| Oliver | Sakowitz | oliver.sakowitz@gmail.com |
| Oliver | Sakowitz | oliver.sakowitz@gmail.com |
| Renan | Sanchez-Porras | renan_md@hotmail.com |
| Oddrun | Sandrød | Oddrun.Sandrod@stolav.no |
| Kari | Schirmer-Mikalsen | kari.schirmer-mikalsen@ntnu.no |
| Kari | Schirmer-Mikalsen | kari.schirmer-mikalsen@ntnu.no |
| Rico Frederik | Schou | [rico@mymedic.dk](mailto:rico@mymedic.dk) |
| Charlie | Sewalt | c.sewalt@erasmusmc.nl |
| Peter | Smielewski | ps10011@cam.ac.uk |
| Abayomi | Sorinola | sorinola_abayomi@hotmail.com |
| Emmanuel | Stamatakis | [eas46@cam.ac.uk](mailto:eas46@cam.ac.uk) |
| Ewout W. | Steyerberg | [e.steyerberg@erasmusmc.nl](mailto:e.steyerberg@erasmusmc.nl) |
| Nino | Stocchetti | stocchet@policlinico.mi.it |
| Nina | Sundström | [Nina.Sundstrom@vll.se](mailto:Nina.Sundstrom@vll.se) |
| Riikka | Takala | [riikka.takala@tyks.fi](mailto:riikka.takala@tyks.fi) |
| Viktória | Tamás | tamas.viktoria@pte.hu |
| Tomas | Tamosuitis | tomas.tamosuitis@kaunoklinikos.lt |
| Olli | Tenovuo | olli.tenovuo@tyks.fi |
| Matt | Thomas | Matt.Thomas@nbt.nhs.uk |
| Dick | Tibboel | d.tibboel@erasmusmc.nl |
| Christos | Tolias | christos.tolias@nhs.net |
| Tony | Trapani | tony.trapani@monash.edu |
| Cristina Maria | Tudora | cristina.tudora@neuromed.ro |
| Andreas | Unterberg | Andreas.Unterberg@med.uni-heidelberg.de |
| Peter | Vajkoczy | Peter.Vajkoczy@charite.de |
| Egils | Valeinis | Egils.Valeinis@latnet.lv |
| Shirley | Vallance | S.Vallance@alfred.org.au |
| Zoltán | Vámos | azozoka@gmail.com |
| Gregory | Van der Steen | gregory@webstone.be |
| Jeroen T.J.M. | van Dijck | j.van.dijck@haaglandenmc.nl |
| Thomas A. | van Essen | T.A.van_Essen@lumc.nl |
| Roel | van Wijk | roel-van-wijk@ziggo.nl |
| Alessia | Vargiolu | neurorianimazione@hsgerardo.org |
| Emmanuel | Vega | emmanuel.vega@chru-lille.fr |
| Anne | Vik | [anne.vik@ntnu.no](mailto:anne.vik@ntnu.no) |
| Anne | Vik | [anne.vik@ntnu.no](mailto:anne.vik@ntnu.no) |
| Rimantas | Vilcinis | rimantas.vilcinis@kaunoklinikos.lt |
| Victor | Volovici | v.volovici@erasmusmc.nl |
| Peter | Vulekovic | pvulekovic@gmail.com |
| Eveline | Wiegers | e.wiegers@erasmusmc.nl |
| Guy | Williams | gbw1000@wbic.cam.ac.uk |
| Stefan | Winzeck | sw742@cam.ac.uk |
| Stefan | Wolf | stefan.wolf@charite.de |
| Alexander | Younsi | alexander.younsi@med.uni-heidelberg.de |
| Frederick A. | Zeiler | [umzeiler@myumanitoba.ca](mailto:umzeiler@myumanitoba.ca) |
| Agate | Ziverte | agate.ziverte@inbox.lv |
| Tommaso | Zoerle | tommaso.zoerle@policlinico.mi.it |
