# Supplementary material for: Pharmaceutical Venous Thrombosis Prophylaxis in Critically Ill Traumatic Brain Injury Patients
Source: Neurotrauma Rep. 2022 Jan 7;2(1):4–14. doi: 10.1089/neur.2021.0037 (PMC8804253; doi:10.1089/neur.2021.0037)
Supplement: Supplemental data [file Supp_FileS1.docx]

Supplemental file 1

| Table 1. Baseline characteristics of patients receiving pVTE prophylaxis in centers using more pVTE prophylaxis versus those using less pVTE prophylaxis. | | | | | |
| --- | --- | --- | --- | --- | --- |
| N= 1962  (Centres>10 patients) | Non-aggressive centre  Prophylaxis <median%  N= 970 | | Aggressive centre  Prophylaxis >=median%  N= 992 | | P-value |
| Age (median, IQR) | 51 [34-66] | | 52 [32-66] | | 0.960 |
| Gender, male (N,%) | 719 | (74.1) | 723 | (72.9) | 0.568 |
| Mechanical DVT prophylaxis | 266 | (29.4) | 578 | (60.0) | <0.001 |
| ISS (median, IQR) | 26 [20-41] | | 32 [25-43] | | <0.001 |
| GCS  Mild | 354 | (38.9) | 300 | (31.8) | 0.003 |
| Moderate | 130 | (14.3) | 172 | (18.3) |  |
| Severe | 427 | (46.9) | 470 | (49.9) |  |
| CT (N,%)  tSAH | 647 | (74.7) | 624 | (74.2) | 0.851 |
| EDH | 160 | (18.4) | 161 | (19.1) | 0.754 |
| Contusion | 492 | (56.7) | 475 | (56.3) | 0.927 |
| Marshall (N, %) |  |  |  |  | 0.006 |
| I | 91 | (10.5) | 94 | (11.1) |  |
| II | 436 | (50.2) | 359 | (42.5) |  |
| III | 74 | (8.5) | 66 | (7.8) |  |
| IV | 9 | (1.0) | 17 | (2.0) |  |
| V/VI | 259 | (29.8) | 308 | (36.5) |  |
| Preinjury ASA (N, %)  1) Normal healthy | 514 | (56.9) | 522 | (54.5) | 0.007 |
| 2) Mild systemic disease | 278 | (30.8) | 340 | (35.5) |  |
| 3) Severe systemic | 98 | (10.8) | 92 | (9.6) |  |
| 4) Severe systemic constant threat to life | 14 | (1.5) | 3 | (0.3) |  |
| Cause of injury (N, %)  Road traffic incident | 368 | (39.8) | 465 | (48.7) | 0.001 |
| Incidental fall | 416 | (45.0) | 364 | (38.2) |  |
| Violence/assault | 40 | (4.3) | 42 | (4.4) |  |
| Suicide attempt | 28 | (3.0) | 16 | (1.7) |  |
| Other | 72 | (7.8) | 67 | (7.0) |  |
| General VTE risk factors (N, %)  BMI>25 | 389 | (54.1) | 430 | (53.4) | 0.828 |
| History of VTE | 7 | (0.7) | 12 | (1.2) | 0.383 |
| Central venous catheter | 375 | (39.6) | 423 | (42.8) | 0.159 |
| Invasive blood pressure | 747 | (78.8) | 862 | (87.2) | <0.001 |
| Cranial Surgery | 319 | (32.9) | 442 | (45.0) | <0.001 |
| Extracranial surgery | 242 | (25.0) | 317 | (32.2) | <0.001 |
| Comorbidity ^1^ | 178 | (18.4) | 194 | (19.6) | 0.533 |
| Length of ICU stay | 6 [2-14] |  | 7 [2-16] |  | 0.167 |
| Length of hospital stay | 13 [7-28] |  | 16 [7-31] |  | 0.036 |
| Prior medication (N, %)  Anticoagulants | 48 | (5.4) | 52 | (5.5) | 0.957 |
| PAI | 91 | (10.3) | 101 | (10.8) |  |
| Both | 7 | (0.8) | 9 | (1.0) |  |
| This table shows the baseline characteristics of TBI patients admitted to an aggressive centre (ICU) or non-aggressive centre. Aggressiveness is based on more patients than the median percentage patients receiving pharmaceutical prophylaxis in that centre. The median percentage of use in centres is 65%, aggressive centres used more than this median percentaged compared with non-aggressive centres.  1) cardiac (arrhythmia, valvular heart disease, congenital heart disease, thromboembolic heart disease, ischemic heart disease), renal (renal insufficiency or failure), oncologic, hepatic, or sickle cell disease  ASA: American Society of Anesthesiologists, EDH: Epidural hematoma, ISS: Injury Severity Scale, PAI: platelet aggregation inhibitors, tSAH: traumatic subarachnoid hemorrhage VTE: venous thromboembolism | | | | | |
