# Supplementary material for: Pharmaceutical Venous Thrombosis Prophylaxis in Critically Ill Traumatic Brain Injury Patients
Source: Neurotrauma Rep. 2022 Jan 7;2(1):4–14. doi: 10.1089/neur.2021.0037 (PMC8804253; doi:10.1089/neur.2021.0037)
Supplement: Supplemental data [file Supp_FileS2.docx]

Supplemental file 2


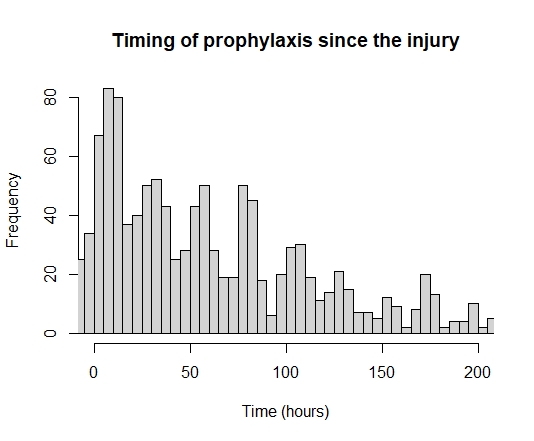


Figure 1. This figure shows the variation in start time of pVTE prophylaxis after the injury. The x-axis shows the time in hours since the injury and the y-axis shows how many patients received pVTE prophylaxis at that starting time.
